# VCAN is upregulated in the cervical cancer stroma and modulates macrophage polarization

**DOI:** 10.3389/fonc.2026.1902341

**Published:** 2026-08-28

**Authors:** Shijia Liu, Tongguo Yang, Alan Chu, Chen Sun, Ting Chai, Mengxi Li, Zongwen Liu, Mengfan Zhang

**Affiliations:** 1 Department of Radiation Oncology, The Second Affiliated Hospital of Zhengzhou University, Zhengzhou University, Zhengzhou, China; 2 Department of Interventional Radiology, The First Affiliated Hospital of Zhengzhou University, Zhengzhou University, Zhengzhou, China

**Keywords:** cervical cancer, macrophage, single-cell RNA sequencing, tumor microenvironment, versican

## Abstract

The extracellular matrix (ECM) is a major component of the tumor microenvironment (TME) and plays an important role in cervical cancer progression. Versican, encoded by the VCAN gene, is a member of the aggrecan/proteoglycan family and has been reported as a marker of poor prognosis in cervical cancer. However, the versican-producing cells in cervical cancer tissue are not clearly identified. To identify the versican-producing cells in cervical cancer tissue, single-cell RNA sequencing (scRNA-seq) data of cervical cancer tissue are re-analyzed, and we find that versican is highly expressed in monocytes and fibroblasts. Immunohistochemical staining of cervical cancer and adjacent normal tissues showed that versican was predominantly expressed in non-parenchymal cells. Multiplex immunofluorescence staining further identified versican-expressing macrophages in cervical cancer tissues. We subsequently collected peripheral blood mononuclear cells (PBMCs) from a patient with cervical cancer before and after radiotherapy and performed scRNA-seq to investigate the highly variable genes in PBMCs. Notably, VCAN was identified as a monocyte-enriched gene and showed relatively high expression in the patient’s circulating monocytes. We further investigated the effect of Versican core protein (VCAN-V3) on macrophages. The results suggest that VCAN-V3 predominantly suppresses M1-associated gene expression and induces a partial shift toward an M2-like transcriptional phenotype in RAW264.7 cells. In conclusion, our study demonstrates that stromal VCAN is upregulated in cervical cancer tissue. VCAN-V3 may influence the tumor microenvironment by modulating macrophage polarization-associated responses.

## Introduction

Cervical cancer is one of the most common malignancies among women worldwide (1). For early-stage and locally invasive cervical cancer, the treatment includes surgery and concurrent chemotherapy and radiation therapy (1, 2). Concurrent chemoradiotherapy is the standard of care for most stages of locally advanced cervical cancer. However, more than half of patients who receive concurrent chemoradiotherapy will develop recurrent disease or distant metastasis (2). The extracellular matrix (ECM) is an important component of the cellular microenvironment. Cancer cells are surrounded by ECM. ECM components are essential for cellular signal transduction and play an important role in the carcinogenesis and progression of cancer tissue (3). Therefore, a better understanding of the ECM in cervical cancer is helpful for predicting and monitoring the effects of clinical therapy.

Versican (VCAN) is a member of the aggrecan/proteoglycan family. Most of its isoforms consist of an N-terminal G1 domain, a glycosaminoglycan (GAG) attachment region and a C-terminal G3 domain (4). Versican is proved to be involved in cancer development via regulating tumor-promoting inflammation, immune surveillance evasion, and immunomodulation (5). Previous studies have revealed that Versican protein is increased in cervical cancer tissue and positively correlated with poor clinical prognosis (4, 6). Versican was determined to be expressed in the myofibroblasts of stroma in cervical cancer in a previous study (6). Recently, it has been demonstrated that in addition to myofibroblasts, other cells including glioma cells, adipocytes, and monocytes, produce versican as well (7–9). Therefor the myofibroblasts may not be the only producing cell in the cervical cancer tissue. Thus, identifying the versican-producing cells of cervical cancer may contribute to a better understanding of its tumor microenvironment (TME).

Single-cell RNA sequencing (scRNA-seq) has been widely used to identify subsets and distinct markers of all sorted cells in the tissue for its remarkable resolution of transcriptome in each cell. A few studies have screened the subclusters and identified distinct markers of those cells in the cervical cancer tissue (10–12). Spatial multi-omic analysis further showed that cancer-associated fibroblasts contribute to distinct immunoregulatory ecosystems in cervical squamous cell carcinoma (13). However, the versican-producing cells are not focused on the results of those studies. In this study, we analyze the single-cell RNA sequencing data of cervical cancer tissue and peripheral blood mononuclear cells (PBMCs) from patients to determine the versican-producing cells and find a proportion of can-producing cells are macrophages. Meanwhile, VCAN-positive macrophages are identified with immune staining of human cervical cancer tissue. Furthermore, VCAN-V3 predominantly suppresses M1-associated gene expression and induces a partial shift toward an M2-like transcriptional phenotype in RAW264.7 cells. These findings suggest that VCAN-V3 may modulate the cervical cancer microenvironment.

## Materials and methods

### Cell culture

RAW264.7 cells and 293T cells were kindly provided by the Shanghai Biochemistry and Cell Biology Cell Bank of the Chinese Academy of Sciences. Cells were cultured in Dulbecco’s Modified Dulbecco’s Medium (DMEM) supplemented with 10% heat inactivated fetal bovine serum (Thermo Fisher Scientific), and antibiotics (100 U/mL penicillin and 10 µg/mL streptomycin) in an incubator containing 5% CO2 at 37°C. The mycoplasma was regularly detected with performed regularly with a PCR kit (C0301S, Beyotime, China). The culture cells used in all experiments were free of mycoplasma contamination.

### Lentiviral vector construction and transfection

The cDNA of mouse Vcan transcript variant 4 (NM_001134475.2) was synthesized by Sangon Biotech (China). This transcript encodes NCBI protein isoform 4, corresponding to the conventional VCAN-V3 isoform. The cDNA was cloned into the PLVX-EF1A-MCS-WPRE-CMV-EGFP:T2A:Puro plasmid using a seamless cloning kit (D7010M, Beyotime, China). The purified Vcan over-expression plasmid and control vector plasmid with pCMV-VSV-G and pCAG-dR8.9 were co-transfected into 293T cells for virus packing with BeyoPEI™ transfection reagent (Beyotime, China). The virus supernatant was centrifuged and filtered with 0.45um syringe filters and then used for transfection. The culture cells were transfected with virus solution mixed with polybrene (Beyotime, China). Then cells were incubated with 2μM puromycin (Beyotime, China) for selection of stable transfected cells.

### Human PBMC isolation and scRNA-seq

PBMCs were collected and sequenced from a cervical cancer patient with ethical approval from the Second Affiliated Hospital of Zhengzhou University (2023009). The written informed consent was obtained before the patient entered the research, and there was no undue influence on the patient to consent. The PBMCs were isolated from the whole blood of the patient. Briefly, PBMCs were isolated with the Human Peripheral Blood Lymphocyte Separation Medium kit (C0025, Beyotime Biotech. Inc., China) followed by the manufacturer’s protocol. The isolated PBMCs with a viability above 80% were used for scRNA-seq. The library construction, sequencing and data analysis were performed by Hangzhou KaiTai Biotechnology Co., Ltd. The in-house original sequencing data were deposited in the Genome Sequence Archive for Human (GSA-Human) platform (https://ngdc.cncb.ac.cn/gsa-human/) with an accession number. The scRNA-seq data from human cervical cancer tissue were collected from GSE197461 at Gene Expression Omnibus (GEO) database (14).

### Single-cell RNA-sequencing data processing and bioinformatic analysis

Raw sequencing data from the in-house PBMC samples were processed using the Cell Ranger count pipeline (10x Genomics). Sequencing reads were aligned to the human GRCh38 (hg38) reference genome using STAR, and gene-by-cell unique molecular identifier (UMI) count matrices were generated. Downstream analyses were performed in R using Seurat v4 (15). Genes detected in fewer than three cells were excluded, and cells with more than 200 but fewer than 6,000 detected genes and a mitochondrial transcript proportion below 15% were retained for subsequent analysis. Gene-expression data were normalized using SCTransform, and 2,000 highly variable genes were selected for principal component analysis. The first 30 principal components were used for shared-nearest-neighbor graph-based clustering, and uniform manifold approximation and projection (UMAP) was used for visualization. Cell identities were initially assigned using the SingleR package and subsequently verified manually according to established cell-type-specific marker genes (16). Cluster marker genes were identified using the FindAllMarkers function in Seurat by comparing each cluster with all remaining cells using the Wilcoxon rank-sum test, with logfc.threshold = 0.25 and min.pct = 0.1. P values were adjusted for multiple comparisons using the Bonferroni method. The GSE197461 dataset was independently reanalyzed using the same downstream Seurat workflow for normalization, dimensionality reduction, clustering, and cell-type annotation.

### Quantitative real-time polymerase chain reaction

Gene expression levels were quantified by real-time reverse transcription polymerase chain reaction (RT-qPCR). Total mRNA was isolated from cells using Tri-reagent (Sigma Aldrich) according to the manufacturer’s protocol. Concentration of RNA was determined by Nano-Drop 2000c (Thermo Fisher Scientific). cDNA was synthesized with 2µg RNA template by HiScript 1st Strand cDNA Synthesis Kit (Vazyme, China). Gene expression was quantified by primers with SybrGreen kit (Vazyme, China) by real-time polymerase chain reaction on the QuantStudio 3 system (Thermo Fisher Scientific). The primers are listed in **Table 1**. All experimental conditions were processed in parallel within each independent experiment. Samples were analyzed in technical duplicate, and the mean Ct value was used for subsequent calculations. Relative gene expression was calculated using the 2^−ΔΔCt method, with *Rps18* used as the housekeeping gene. The experiments were independently repeated three times.

**Table 1:** Primers used in the RT-qPCR.

Specie	Gene	Forward primer	Reverse primer
Mouse	Rps18	TGGGAAGTACAGCCAGGTTC	AGTGGTCTTGGTGTGCTGAC
	Vcan	CCATGCACTACATCAAGCCAA	TGGGTGGTAAGGTAGGTAAGGT
	Arg1	GGAACTGAAAGGAAAGTTCCCA	TCGCAAGCCAATGTACACGA
	Nos2	GGTGAAGGGACTGAGCTGTT	ACGTTCTCCGTTCTCTTGCAG
	Tnf	CCCAAAGGGATGAGAAGTTCC	GCTCCTCCACTTGGTGGTT
	Mrc1	TTCAGCTATTGGACGCGAGG	GAATCTGACACCCAGCGGAA
	Cd86	CAGCACGGACTTGAACAACC	CTCCACGGAAACAGCATCTGA

### Immunohistochemistry staining and multiplex immunofluorescence staining

The paraffin-embedded sections were collected and processed with ethical approval from the Second Affiliated Hospital of Zhengzhou University (2023009). The histological analyses included paired cervical cancer and adjacent noncancerous tissue samples from 10 patients who underwent radical surgery. The available clinicopathological characteristics of these patients are presented in **Supplementary Table 1**. The sections were deparaffinized and performed with the procedures of blocking endogenous peroxidase activity and antigen retrieval. The sections were blocked with 3% BSA (Servicebio, China) and then incubated with diluted primary antibody solution overnight at 4°C. Washed by PBS for 3 times, the sections were incubated with diluted HRP-conjugated secondary antibody (Servicebio, China) for 1 hr at room temperature. After being washed, the sections were incubated with DAB substrate solution (Servicebio, China). Afterwards, the sections were counterstained with hematoxylin for 1–2 min and then dehydrated and mounted with mounting medium (Servicebio, China). The multiplex immunofluorescence staining was performed with a staining kit according to the manufacturer’s protocol. The sections were blocked and then incubated with diluted primary antibody solution, diluted corresponding HRP-conjugated secondary antibody (Servicebio, China) and corresponding TSA solution (Servicebio, China) in sequence. The procedures were repeated for 3 times to stain each antigen and then stained with DAPI solution (Servicebio, China), incubated with quenching agent (Servicebio, China) and anti-fluorescence quenching sealing tablets (Servicebio, China). All the slides were scanned with Pannoramic scanner (3DHISTECH). The primary antibodies used for staining included anti-VCAN (ab270445, Abcam), anti-COL1A1 (PA5-29569, Thermo Fisher Scientific) and anti-CD68 (GB113150-100, Servicebio).

### Statistical analysis

Statistical analyses were performed using GraphPad Prism version 10 (GraphPad Software; Dotmatics). Quantitative data are presented as the mean ± standard deviation (SD), with individual data points shown. The value of *n* represents the number of independent biological experiments or patient-matched pairs, as specified in the figure legends. Comparisons between two matched groups were performed using two-tailed paired Student’s *t*-tests. Experiments involving two factors were analyzed using two-way repeated-measures analysis of variance (ANOVA), followed by Šídák’s multiple-comparisons test. A *P* value <0.05 was considered statistically significant. Statistical significance is indicated as follows: ns, *P*≥0.05; *P < 0.05; **P < 0.01; ***P < 0.001; and ****P < 0.0001.

## Results

**Figure 1:**
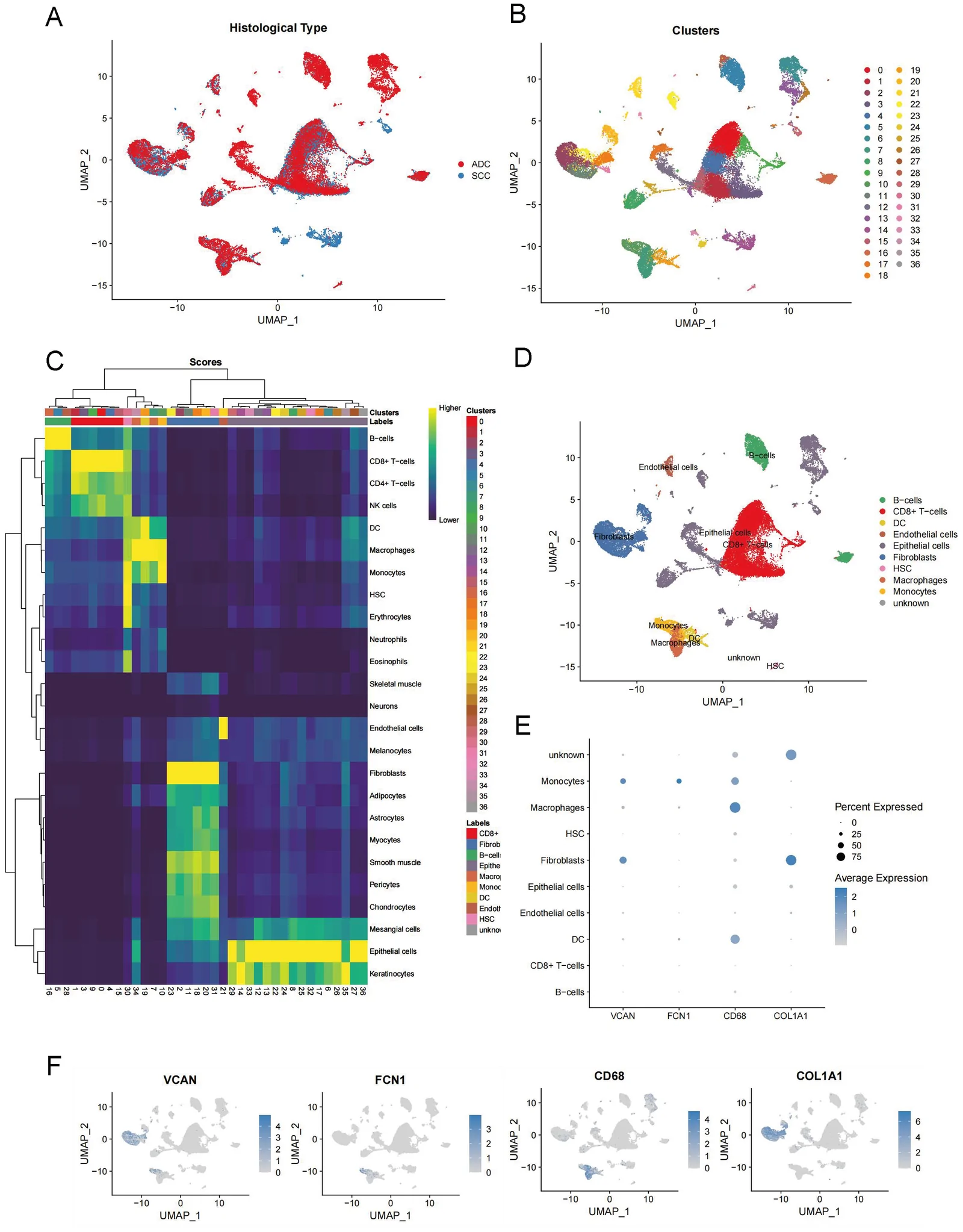
7Versican is expressed in fibroblasts and monocytes in cervical cancer. **(A)** The integrated scRNA-seq data of adenocarcinoma (ADC) and squamous cell carcinoma (SCC) of cervical cancer. **(B)** The cluster classification of scRNA-seq data. **(C)** The cell annotation score of each cluster. **(D)** The dimplot of annotated cell types. **(E)** The expression of interested genes including VCAN, FCN1, CD68 and COL1A1 shown as dotplot. **(F)** The expression of interested genes including VCAN, FCN1, CD68 and COL1A1 shown as featureplot.

The scRNA-seq data of human cervical cancer tissues were re-analyzed, shown as **Figure 1A**. All the cells were clustered as 37 subsets, shown as **Figure 1B**. To determine the cell types of each cluster, the data were processed with automatic annotation algorithm. The annotation scores were shown as **Figure 1C**. Each cluster was labeled with the cell type of highest score and plotted in **Figure 1D**. FCN1 was identified as an infiltrating macrophage marker. CD68 and COL1A1 are established macrophage markers and fibroblast markers, respectively. Therefore, FCN1, CD68 and COL1A1 were selected as markers of infiltrating monocytes, macrophages, and fibroblasts, respectively. And the average expression of VCAN in each cell type is shown as **Figure 1E**. Meanwhile, the expression of VCAN, FCN1, CD68 and COL1A1 was plotted in the featureplot and the results demonstrated the expression of VCAN was high in fibroblasts and infiltrating monocytes, shown as **Figure 1F**. Taken together, the scRNA-seq data of cervical cancer tissue revealed the VCAN-producing cells were mainly fibroblasts and infiltrating monocytes.

**Figure 2:**
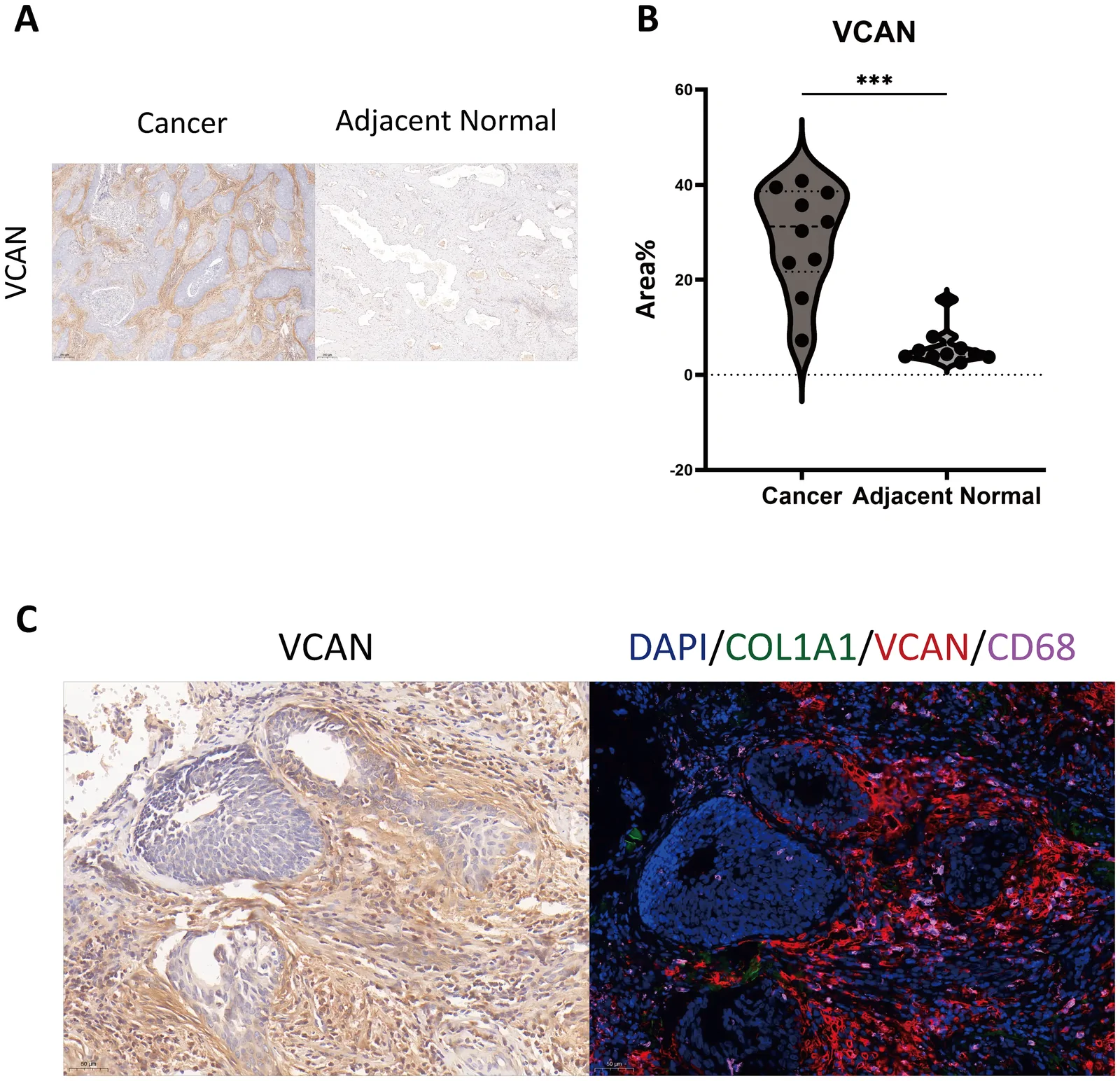
VCAN is upregulated in the stromal compartment of cervical cancer tissue. **(A)** The immunohistochemistry staining of VCAN in the cancer tissue and adjacent normal tissue. Scale bar = 200μm. **(B)** The quantification of VCAN protein positive area between cancer tissue and adjacent normal tissue in patients with cervical cancer (n=10). Data are presented as mean ± SD with individual values shown. Statistical significance was determined using a two-tailed paired Student’s t-test. ***P < 0.001. **(C)** The immunohistochemistry staining of VCAN and multiplex immunofluorescence staining of COL1A1, VCAN and CD68 on the consecutive sections of cervical tissue. Scale bar = 50μm.

To further verify the VCAN-positive cells, the paraffin-embedded sections of human cervical cancer tissue and adjacent noncancer tissue from radical surgery were collected to perform immunohistochemistry, shown as **Figures 2A, B**. The staining results demonstrated that the VCAN protein expression was increased in cervical cancer tissue compared with adjacent normal tissue. In addition, VCAN was predominantly located in the stroma surrounding the cancer cells. According to the previous results of scRNA-seq, VCAN expression was detected in infiltrating monocytes and fibroblasts. To identify the VCAN-producing cells, the consecutive paraffin sections of cervical cancer tissue were collected and stained with immunohistochemistry and multiplex immunofluorescence, respectively, shown as **Figure 2C**. The multiplex immunofluorescence images showed that VCAN signals were detected in both CD68-positive macrophages and COL1A1-positive fibroblasts, consistent with the scRNA-seq findings. Collectively, these findings demonstrate that VCAN is upregulated in the stromal compartment of cervical cancer tissue and suggest that macrophages contribute to stromal VCAN expression.

**Figure 3:**
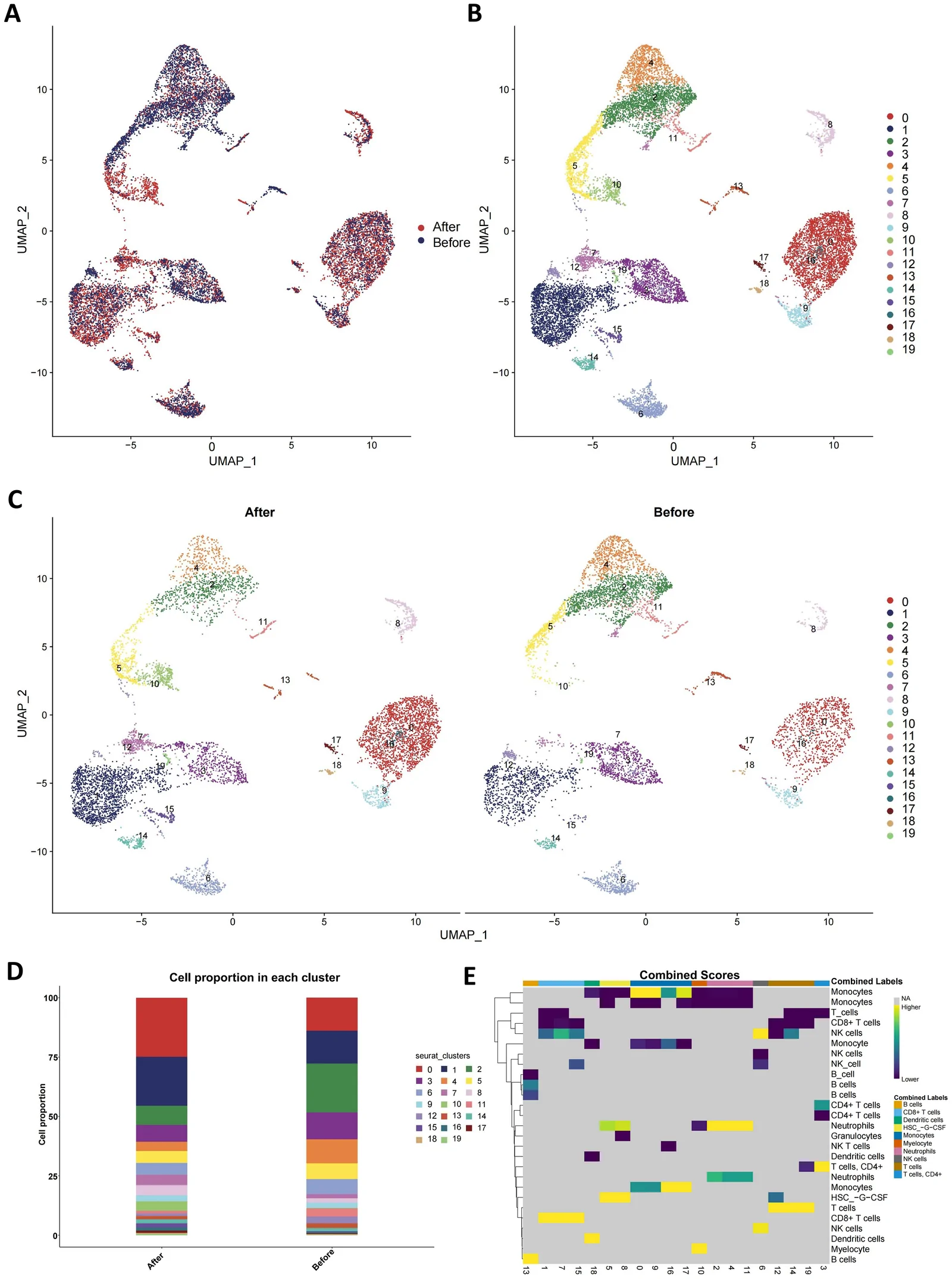
The clusters of PBMCs before and after radiation therapy from a patient with cervical cancer. **(A)** The integrated UMAP plot of all PBMCs. **(B)** The cell clusters of all PBMCs. **(C)** The separate UMAP plot of the cell clusters of the PBMCs. **(D)** The cell proportion of each cluster of the PBMCs before and after radiation therapy. **(E)** The annotation scores of cell clusters of all PBMCs.

Given that infiltrating monocytes expressed VCAN in cervical cancer tissue, we hypothesized that VCAN might also be highly expressed in circulating monocytes and therefore investigated its expression in PBMCs from a patient with cervical cancer. The scRNA-seq data of PBMCs were integrated and processed with UMAP dimension reduction algorithm, shown as **Figure 3A**. The PBMCs are classified into 20 clusters, shown as **Figure 3B**. The separate UMAP plot of the clustered cells indicated that cell proportion of PBMC clusters varied not much before and after radiation therapy, shown as **Figures 3C, D**. Thereafter, the cell clusters were annotated with a combined scoring system and the annotation scores of the cell clusters were displayed as **Figure 3E**. According to the annotation scoring algorithm, the cluster 0, cluster 1 and cluster 2 were monocytes, CD8+ T cells and neutrophils, respectively.

**Figure 4:**
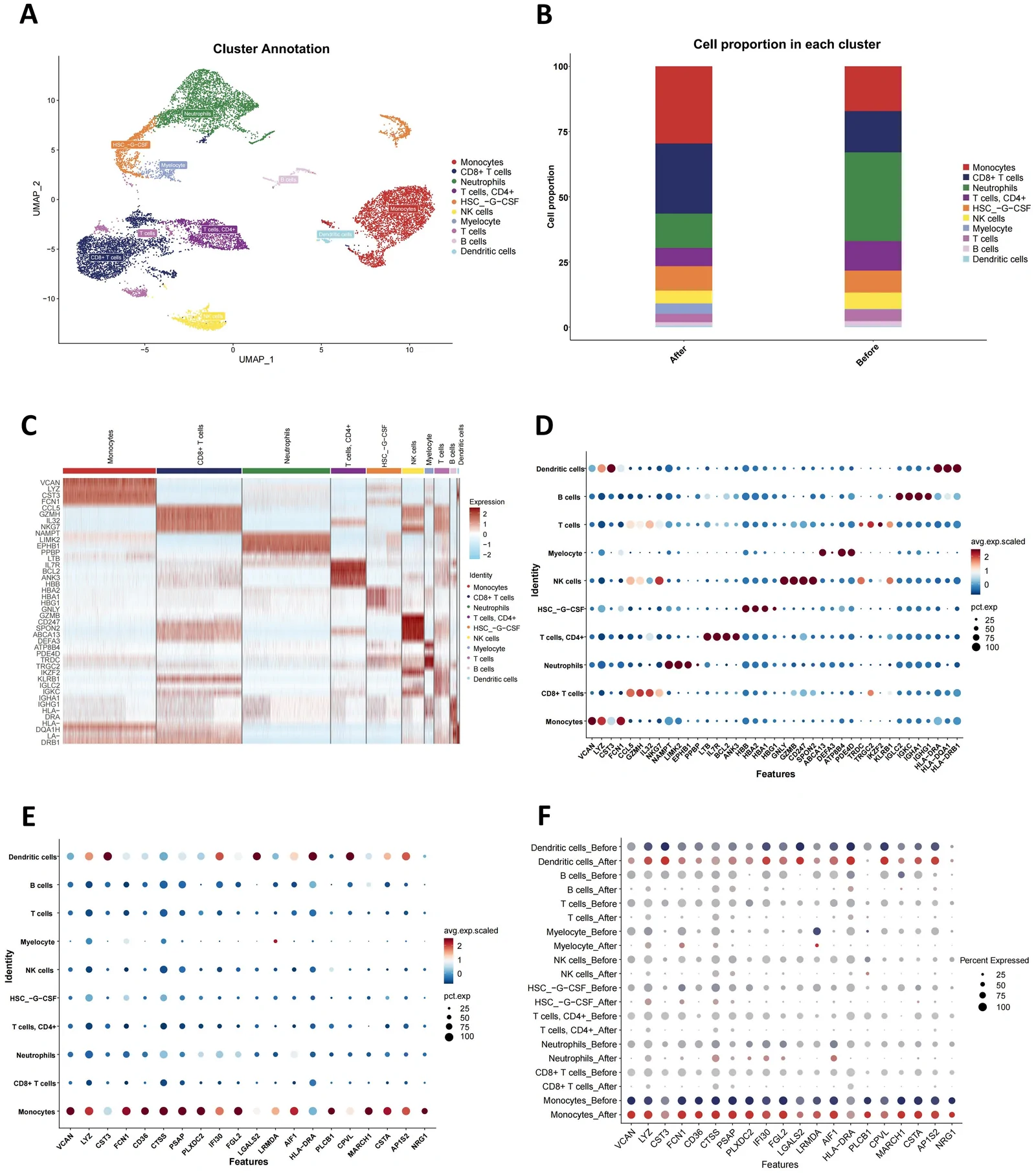
The differentially expressed genes in the monocytes. **(A)** The UMAP plot with annotated cell types in the integrated PBMC data. **(B)** The proportion of each cell type before and after radiation therapy in the PBMCs. **(C)** The heatmap of the marker genes of each cell type in the integrated PBMC data. **(D)** The dotplot of the marker genes of each cell type in the integrated PBMC data. **(E)** The dotplot of highly expressed genes of monocytes across all the cell types in the integrated PBMC data. **(F)** The separate dotplot of highly expressed genes of monocytes across all the cell types before and after radiation therapy.

The cells were combined according to the cell type annotation results and the UMAP plot of annotated cells was shown as **Figure 4A**. In addition, the proportion of cell types before and after radiation therapy was calculated and shown as **Figure 4B**. In this patient, the post-radiotherapy sample showed higher proportions of monocytes and CD8+ T cells and a lower proportion of neutrophils than the pre-radiotherapy sample. The expression profile of marker genes of each cell type was displayed as heatmap and dotplot, respectively, and shown as **Figures 4C, D**. The results revealed the cell types were clearly distinguished by the differentially expressed marker genes. To further investigate the highly expressed distinct genes of monocytes, the top 20 highly variable genes of monocytes were selected and displayed in the dotplot, shown as **Figure 4E**. The separate dotplot of those genes were displayed as **Figure 4F**. The results demonstrated most of the highly variable genes of monocytes had similar expression level before and after radiation therapy. Among the highly variable genes, VCAN, LYZ, FCN1 and CD36 were confined to be highly expressed in the monocytes.

**Figure 5:**
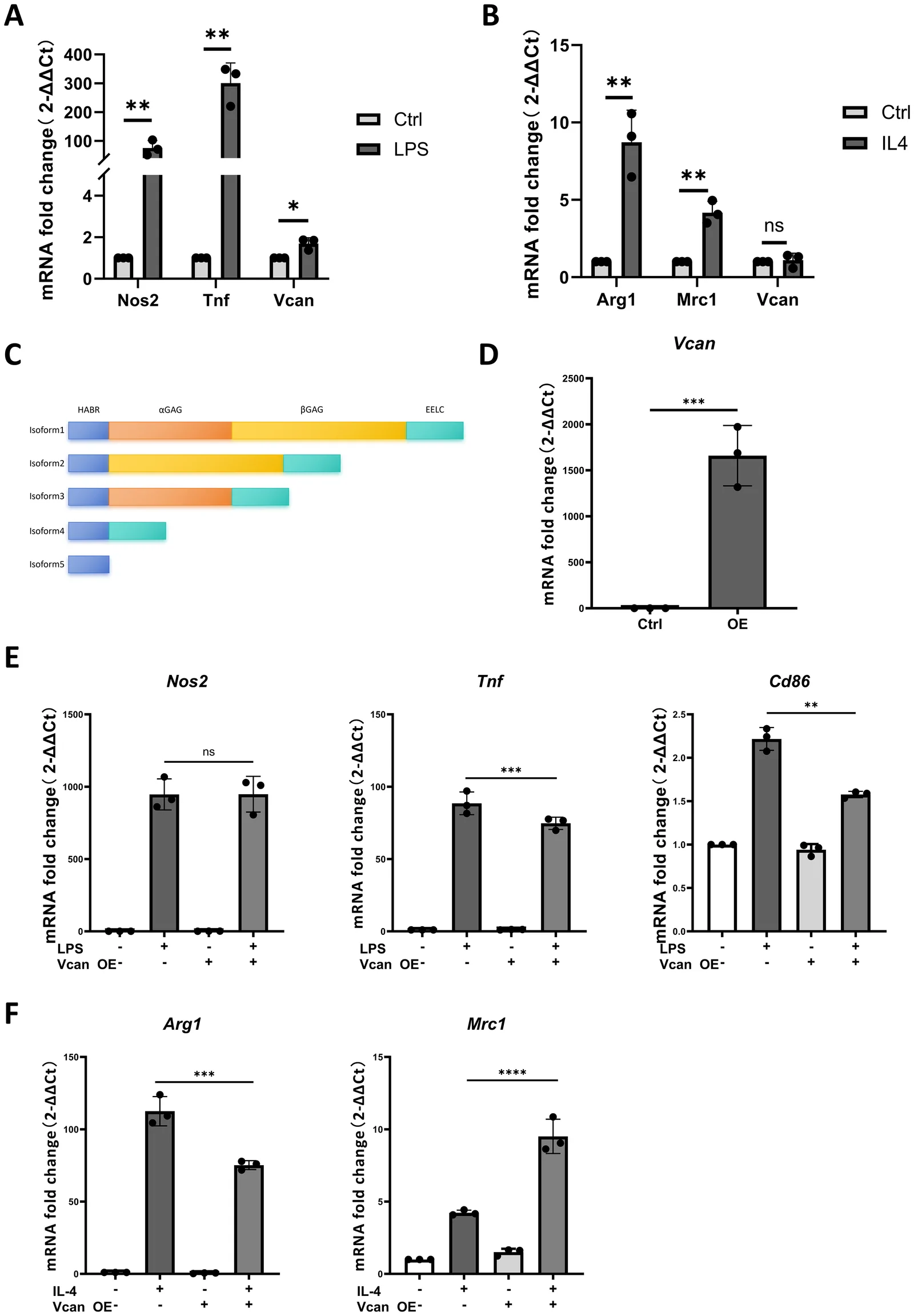
Vcan promotes alternative activation of macrophages. **(A)** The mRNA expression of Nos2, Tnf and Vcan in the LPS stimulated RAW264.7 cells (n=3). The RAW264.7 cells were stimulated with solvent control or 100ng/ml LPS for 4h. **(B)** The mRNA expression of Arg1, Mrc1 and Vcan in the IL-4 stimulated RAW264.7 cells (n=3). The RAW264.7 cells were stimulated with solvent control or 20ng/ml IL-4 for 24h. **(C)** The illustration of protein domains of Vcan isoforms. HABR: hyaluronan binding region; GAG: glycosaminoglycan; EELC: two epidermal growth factor repeats (EE), a lectin binding domain (L) and a complement regulatory region. **(D)** The mRNA expression of Vcan in the lentivirus transduced control RAW 264.7 cells and over-expression RAW264.7 cells (n=3). **(E)** The mRNA expression of Nos2, Tnf and Cd86 in the LPS stimulated control RAW264.7 cells and Vcan over-expression RAW264.7 cells (n=3). **(F)** The mRNA expression of Arg1 and Mrc1 in the IL-4 stimulated control RAW264.7 cells and Vcan over-expression RAW264.7 cells (n=3). Data are presented as mean ± SD, with individual data points shown. The value of *n* represents the number of independent biological experiments, and each data point represents one independent biological experiment. **(A, B, D)** were analyzed using two-tailed paired Student’s *t*-tests. **(E, F)** were analyzed using two-way repeated-measures ANOVA followed by Šídák’s multiple-comparisons test. ns, *P*≥0.05; *P < 0.05; **P < 0.01; ***P < 0.001; and ****P < 0.0001.

Considering macrophages could produce VCAN, we were wondering if the VCAN expression is increased in M1 or M2 macrophages. The murine macrophage cell line RAW264.7 was stimulated by LPS or IL-4 and the M1 and M2 markers were measured by RT-qPCR, respectively. The results indicated that expression of Vcan mRNA was increased in M1 macrophages, shown as **Figures 5A, B**. To further investigate the effect of VCAN on macrophage activation, we considered constructing a stable cell line with high expression of VCAN. The mouse Vcan gene had five different transcript variants and each of them translated into a protein isoform. The protein structure variance was displayed as **Figure 5C**. The over-expression of Vcan (cDNA of mouse VCAN-V3 isoform) was verified by RT-qPCR, shown as **Figure 5D**. The transduced control cells and Vcan over-expression cells were stimulated by LPS and the results revealed that Vcan reduced the mRNA expression of Tnf and Cd86, shown as **Figure 5E**. In parallel, the transduced control cells and Vcan over-expression cells were stimulated by IL-4 and the results revealed that Vcan increased the mRNA expression of Mrc1 but slightly reduced the mRNA expression of Arg1. Collectively, Vcan prevented M1-associated gene expression and promotes a partial M2-like transcriptional shift.

## Discussion

According to the scRNA-seq data and the staining results of cervical cancer tissue, we demonstrate that the VCAN-producing cells include monocytes and fibroblasts in the cervical cancer TME. In addition, exploratory scRNA-seq analysis of PBMCs from the single patient included in this study showed relatively high VCAN expression in circulating monocytes. More importantly, VCAN prevents M1 polarization and promotes a partial M2-like transcriptional shift in macrophages. Our results indicate that VCAN may influence the cervical cancer microenvironment by modulating macrophage polarization.

VCAN is a kind of chondroitin sulfate proteoglycan and is encoded from a single gene locus on chromosome 5q14.3 in humans (5). VCAN is found to be increased and expressed in the surrounding stroma of cervical cancer (4, 6). However, the VCAN-producing cells in cervical cancer tissue are deemed as myofibroblasts according to the results of Gorter et al. (6). Interestingly, in the PBMC dataset from the single patient analyzed in this study, VCAN expression was relatively enriched in circulating monocytes. The result is similar to a previous study, which THP-1 cell, a human monocyte cell line, has been proven to produce VCAN (17). Considering the VCAN is reported to be expressed in other cell types including monocytes and tumor cells (8, 9), our results highlight the infiltrating monocytes may contribute to VCAN expression in the cervical cancer TME. Similarly, a pan-cancer single-cell analysis linked VCAN to monocyte-derived macrophages and identified VCAN, SELL, and FCN1 as genes expressed during the early stages of inferred monocyte-to-macrophage differentiation (18). Tumor-associated macrophages (TAMs) are the most abundant immune cells of the TME, representing about 50% of whole hematopoietic cells (19). TAMs originate from tissue-resident macrophages and infiltrating macrophages. TAMs modulate ECM of TME via expressing MMPs or other molecules to stimulate cancer-associated fibroblasts (CAFs). Our results suggest that macrophages may contribute to the ECM composition of the cervical cancer TME by directly producing ECM components.

Our results suggest that the VCAN-V3 isoform suppresses M1-associated gene expression and promotes a partial M2-like transcriptional shift in macrophages. Notably, Vcan overexpression increased Mrc1 expression but attenuated the IL-4-induced increase in Arg1 expression. Evidence from experimental perturbation models suggests that individual IL-4-responsive genes may be differentially regulated. In IL-4-stimulated Gps2-deficient mouse bone marrow-derived macrophages (BMDMs), Mrc1, Mgl2, Clec7a, and Ptgs1 expression was enhanced, whereas Arg1 expression remained unchanged (20). Arg1 expression is also sensitive to the cytokine context; in Salmonella-infected mouse BMDMs, IL-4 further enhanced Arg1 expression, whereas IFN-γ suppressed it (21). Moreover, single-cell transcriptomic and chromatin analyses of mouse BMDMs showed that cell-cycle-associated plasticity and chromatin states contribute to heterogeneous responses to polarizing cytokines (22). Accordingly, the discordant changes in Mrc1 and Arg1 may reflect differential regulation of individual components of the IL-4-responsive transcriptional program. In addition, Arg1 induction may be further modulated by other cellular or inflammatory responses associated with Vcan overexpression, although the underlying mechanism remains to be determined.

Human VCAN has five isoforms including V0, V1, V2, V3 and V4 (23). Among the isoforms, the V2 is mostly restricted to the central nervous system while the others are expressed in most tissues (5, 24). VCAN is reported to be increased in LPS stimulated mouse BMDMs (25). In contrast, VCAN is also reported to be increased in IL-10 stimulated human macrophages derived from human PBMCs (26). Interestingly, the IL-10 stimulated macrophages play an important role in promoting progression of cervical cancer (27–29). Currently, there is no study clearly comparing the effect of VCAN isoforms. VCAV-V1 isoform is reported to promote inflammation (30). Whereas macrophage-derived VCAN exhibits anti-inflammatory effect in acute pulmonary inflammation (31). The immunosuppressive effect of VCAN positive macrophages is identified in liver cancer (32). However, the isoform of VCAN is not specificized in previous studies. GAG domain of VCAN can bind to a cell surface receptor such as CD44 or RHAMM (receptor for hyaluronan-mediated motility), which is considered as the mechanism of VCAN isoforms (V0, V1 and V2) modulating macrophage polarization (24, 33). The VCAN-V3 isoform contains no GAGs indicating its effect is independent of CD44 or RHAMM (24). The VCAN-V3 isoform derived from arterial smooth muscle cells decreases monocyte adhesion (34). The evidence suggests the effect of VCAN varies by its isoforms. VCAN-V3 is a naturally occurring splice isoform that lacks both GAG attachment regions but retains the functional G1 and G3 domains. The absence of covalently attached GAG chains minimizes potential confounding effects arising from their structural heterogeneity, thereby providing a more defined model for evaluating VCAN core protein activity. Meanwhile, the retained G1 and G3 domains preserve the potential for interactions with hyaluronan and other extracellular matrix components (23, 35). However, the direct effect of VCAN- V3 isoform on macrophage polarization is not clearly reported in previous studies. Our results suggest VCAN-V3 may modulate macrophage polarization-associated immune responses in the cervical cancer TME. The result is in line with the clinical observation of VCAN expression being associated with poor prognosis of cervical cancer (4).

Several limitations of this study should be acknowledged. The PBMC scRNA-seq analysis was based on samples from a single patient; therefore, the findings should be considered exploratory. In addition, the study did not provide direct evidence that circulating VCAN-positive monocytes differentiate into tumor-associated macrophages. Macrophage polarization was evaluated primarily using transcriptional markers, without validation at the protein level. Moreover, whether VCAN directly induces immunosuppression or promotes cervical cancer progression remains to be established through additional functional assays and *in vivo* studies.

In conclusion, our findings show that stromal VCAN is increased in cervical cancer tissue, with expression detected in both macrophages and fibroblasts. Exploratory PBMC scRNA-seq analysis from a single patient further suggested relatively high VCAN expression in circulating monocytes. VCAN-V3 may influence the tumor microenvironment by modulating macrophage polarization-associated responses. Our study provides insight into the cellular sources and potential immunomodulatory role of VCAN in the cervical cancer microenvironment.

## Statement of informed consent and ethical approval

Human peripheral blood mononuclear cells (PBMCs) were collected and sequenced from a cervical cancer patient with ethical approval from the Second Affiliated Hospital of Zhengzhou University (2023009). The written informed consent was obtained before the patient entered the research, and there was no undue influence on the patient to consent. The paraffin-embedded sections were collected and processed with ethical approval from the Second Affiliated Hospital of Zhengzhou University.

## Availability of code

The analysis of the scRNA-seq data was followed by the Seurat instructions using the R software. The authors declared there was no specific unique function used in the analysis of scRNA-seq data.

## Data Availability

The datasets presented in this study can be found in online repositories. The names of the repository/repositories and accession number(s) can be found below: https://ngdc.cncb.ac.cn/gsa-human, HRA007313.
